# Human Activity Helps Prey Win the Predator-Prey Space Race

**DOI:** 10.1371/journal.pone.0017050

**Published:** 2011-03-02

**Authors:** Tyler B. Muhly, Christina Semeniuk, Alessandro Massolo, Laura Hickman, Marco Musiani

**Affiliations:** 1 Faculty of Environmental Design, University of Calgary, Calgary, Alberta, Canada; 2 Department of Geomatics Engineering, University of Calgary, Calgary, Alberta, Canada; 3 Faculty of Veterinary Medicine, University of Calgary, Calgary, Alberta, Canada; University of Maribor, Slovenia

## Abstract

Predator-prey interactions, including between large mammalian wildlife species, can be represented as a “space race”, where prey try to minimize and predators maximize spatial overlap. Human activity can also influence the distribution of wildlife species. In particular, high-human disturbance can displace large carnivore predators, a trait-mediated direct effect. Predator displacement by humans could then indirectly benefit prey species by reducing predation risk, a trait-mediated indirect effect of humans that spatially decouples predators from prey. The purpose of this research was to test the hypothesis that high-human activity was displacing predators and thus indirectly creating spatial refuge for prey species, helping prey win the “space race”. We measured the occurrence of eleven large mammal species (including humans and cattle) at 43 camera traps deployed on roads and trails in southwest Alberta, Canada. We tested species co-occurrence at camera sites using hierarchical cluster and nonmetric multidimensional scaling (NMS) analyses; and tested whether human activity, food and/or habitat influenced predator and prey species counts at camera sites using regression tree analysis. Cluster and NMS analysis indicated that at camera sites humans co-occurred with prey species more than predator species and predator species had relatively low co-occurrence with prey species. Regression tree analysis indicated that prey species were three times more abundant on roads and trails with >32 humans/day. However, predators were less abundant on roads and trails that exceeded 18 humans/day. Our results support the hypothesis that high-human activity displaced predators but not prey species, creating spatial refuge from predation. High-human activity on roads and trails (i.e., >18 humans/day) has the potential to interfere with predator-prey interactions via trait-mediated direct and indirect effects. We urge scientist and managers to carefully consider and quantify the trait-mediated indirect effects of humans, in addition to direct effects, when assessing human impacts on wildlife and ecosystems.

## Introduction

Predator-prey interactions can be represented as a “space race”, where preys try to minimize and predators try to maximize spatial overlap [1]. Prey must identify space where they can obtain sufficient resources to live (e.g. food, water, cover etc.) and avoid predators [2], as well as habitats that might improve escape ability from predators [3]. Conversely, predators can use space based on the abundance of their prey, or track the distribution of prey resources as cues for areas preferred by prey [4].

When predators influence the distribution of prey it is called a trait-mediated or behaviorally-mediated direct effect [5], [6]. Disturbance by humans - such as activity along roads and trails, can also displace wildlife species via trait-mediated direct effects, i.e., by influencing wildlife behavior and distribution through similar mechanisms as predator-prey interactions [7]. At high disturbance levels humans can displace large carnivore predators, even in not-hunted protected populations (e.g., [8], [9]). Trait-mediated direct effects of humans on predator species can then indirectly affect prey species by creating spatial refugia for prey ( e.g., [10], [11]). Some prey species (e.g., moose [*Alces alces*]) even appear to select space close to humans (e.g., roads) in areas where predator (e.g., grizzly bear [*Ursus arctos horribilis*]) densities are high as a means to avoid encounters with human-avoiding predators [12]. Such trait-mediated direct and indirect effects of humans may apply to whole predator and prey guilds if several predator species are influenced by humans. Human activity may ultimately tip the predator-prey “space race” in favour of prey when humans negatively directly affect predators, which then indirectly has a positive effect on prey.

Trait-mediated indirect effects of humans can potentially have as important implications for wildlife species conservation and management as direct effects of humans. They may create, enhance, ameliorate, or even reverse direct interactions between species [13]. For example, predator displacement by humans can provide refugia for endangered prey species [14]. Our study directly examined, in a spatially-explicit context, the effect of humans on the spatial overlap between large mammalian predator and prey species via trait-mediated direct and indirect effects.

Our research purposes were to: (1) measure occurrence of large mammals at camera traps placed along roads and trails; (2) test whether predators were co-occurring with prey (i.e., predators maximizing spatial overlap), or prey were avoiding predators (i.e., prey minimizing spatial overlap); and (3) test what factor(s), including human presence, predators, food resources (i.e., forage for herbivore prey, prey counts for predators) and habitat (i.e., forest cover and elevation) were influencing predator and prey species occurrence. We used cluster and ordination analyses to describe species co-occurrence (including wild large mammals, humans and cattle) and regression tree analysis to test which factors influenced predator and prey species occurrence. Large terrestrial carnivores are generally sensitive to human disturbance [15], [16], therefore we hypothesized that predator species would avoid high-human use roads and trails. Conversely, humans can provide security cover from predation if prey are less sensitive to human disturbance than predators (see above). In addition, humans might provide food to herbivores through habitat enhancements (e.g., in agricultural fields and around roads and trails [17], [18]). Therefore we predicted that camera sites with high-human use would have higher counts of prey species and lower counts of predators.

## Methods

### Study Area

The study occurred within a montane ecosystem along the eastern slopes of the Rocky Mountains in southwest Alberta, Canada. The extent of the study area was defined by wolf and elk home ranges, calculated from a 95% kernel density estimator of telemetry location data collected from 2004–2007 [19]. The continental divide, i.e., the Alberta-British Columbia provincial border, bounds the western edge of the study area. Towards the east, topography is less rugged, with rolling foothills that eventually level to flat prairie and agricultural lands. Forested lands generally occur in the western half of the study area and open into grasslands to the east.

There were several small towns (populations of 300 to 4,000 people) located within the study area. Lands to the west were predominantly public and to the east were predominantly private. Prevailing land uses included agriculture (primarily livestock production), forestry, natural gas development and recreational activities (e.g., camping and off-highway vehicle use). Cattle (*Bos taurus*) were the dominant domestic herbivore and numbered in the 10,000 to 100,000's [20]. The study area encompassed four wolf (*Canis lupus*) pack home ranges [19], [21], 51 grizzly bears [22], 1,042 elk (*Cervus elaphus*) [23] and an unknown number of cougars (*Puma concolor*), black bears (*Ursus americanus*), coyotes (*Canis latrans*), white-tailed deer (*Odocoileus virginianus*) and mule deer (*O. hemionus*).

### Detecting Species Occurrence Using Camera Traps

To measure human, cattle and wildlife species occurrence, we deployed 43 digital camera traps (RECONYX Silent Image™ Model RM30, RECONYX© Inc., Holmen, WI, USA). As the primary objective of the study was to document relative occurrence of species where humans could also occur, areas around road and trails were ideal locations. Our rationale relies on using an appropriate means for assessing relative densities of large mammals at different sites, which constitute pseudo-experimental replicates receiving different human-use levels. Measuring the density and distribution of several wide-ranging large mammalian species is challenging because it can be expensive, invasive (if animals are captured, such as with telemetry studies) and labour intensive [24], [25]. Recently, digital cameras traps have been used as a relatively inexpensive and non-invasive means to measure large mammal abundance and distribution [26], [27]. Furthermore, digital camera traps are indiscriminate, thus they can provide information about several wildlife species in a region (e.g., [28], [29]) and they can be used to measure the density and distribution of humans too. Permission to use trail cameras was obtained from the University of Calgary Conjoint Faculties Research Ethics Board (CFREB File # 5144).

Comparing use of space by multiple species based on data from camera traps requires some caution, as it can be biased towards larger [30] and more gregarious species [31] that might be more easily detected. Nevertheless, in general the rate of photographing an animal at camera traps is correlated with animal abundance and thus provides a useful index of species occurrence at a location [32], [33], but see [34]. Such an index should be sufficient for comparing species co-occurrence at multiple camera sites collected during the same period within the same study area.

It should be clearly noted here that the study did not rely on absolute measures of density for any species. Obtaining species density information would require a different methodological approach that accounts for habitat and sightability biases (e.g., [28]). This study relies on relative indices of abundance independently gathered for each study species among camera sites. Our methodological approach should allow comparing counts of a given species at a given camera site to counts of the same species at other camera sites. Unbiased sampling was achieved by producing 43 random points within the study area using Hawth's Tools [35] in ArcGIS 9.2 (ESRI© Inc., USA) and placing a camera within 5-meters of the nearest road or trail to each point, ensuring that the view included the area from the camera to the road/trail and an equal area at the other side. To ensure cameras were placed where predator and prey species could potentially interact, random points were generated within the extent of overlapping wolf (i.e. a dominant wild predator species) and elk (i.e. a dominant wild prey species) kernel home range boundaries determined from telemetry data collected in the study area [19]. Cameras were set to the highest sensor sensitivity with a delay of one picture per second and strapped to trees using bungee cords and cable locks at a one meter height facing the trail/road. Cameras were deployed from 17 April to 21 November 2008 for 7,421 trap days (mean = 173 trap days/camera). Thus, cameras measured the “summer” distribution of animals only. Cameras were re-visited at one-month intervals to download data from memory cards, change batteries and replace desiccant packs.

We used a random sampling design to deploy cameras (within the areas determined as explained above), which might have missed sub-areas with especially high or low predator or prey species densities or habitats where predator-prey interactions occur disproportionately. A stratified design based on, for example, predicted density of predator and prey species, or predicted habitat where predator-prey interactions occur, might have influenced the results by providing data across a gradient of species densities and habitat types. However, our research included multiple predator and prey species with different densities and habitat preferences (i.e., we did not have *a priori* knowledge of the density of all species or their habitat preferences), thus a randomized sampling scheme was deemed more appropriate and prone to less bias.

Detected species, and date and time of detection were recorded for each picture taken. If multiple individuals were captured within a single photograph, each individual was counted singularly. Multiple photographs within a short period of time (15 minutes) that were obviously of the same animal were counted as one record, as suggested by other camera trap studies (e.g., [36]). Indices of relative abundance (i.e., photographic rates) were calculated for each species to assess species occurrence at each site. Abundance was indexed as the number of independent captures of each species per 100 trap-days.

### Measuring Habitat at Camera Trap Sites

We used Geographic Information Systems (GIS) data to measure habitat characteristics that might be important to large mammalian species occurrence, including elevation and the amount of high-quality forage habitat and forest cover in the area surrounding camera sites. From a digital elevation model (DEM) we calculated the average elevation within a 1-km radius of each camera site.

To calculate the amount of high-quality forage habitat available to herbivores, first we collapsed a 30-m^2^ spatial resolution 16-class vegetation cover GIS dataset [37] into two forage food-quality classes (high and low [38]) and calculated the area of high-food-quality forage habitat within a 1-km radius of each camera. Second, we obtained a 250-m^2^ spatial resolution dataset of the maximum Normalized Difference Vegetation Index (NDVI) measured during the 2005 growing season. NDVI is an index of vegetation biomass (i.e., forage quantity) that is useful to monitor the effect of vegetation on animals at large scales [39]. As a measure of forage quantity at each location, we calculated the mean of the maximum NDVI value in a plant-growing season within a 1 km radius of each camera. We multiplied this value by the area of high-quality forage habitat to obtain an index of forage quality and quantity (hereafter, referred to as “forage”) within 1 km of each camera. To assess the amount of security cover available to animals around camera sites, we further used the vegetation cover GIS dataset to calculate the amount of actual forest within a 1-km radius of each camera. Overall, classification accuracy of the vegetation map was 80%, as calculated from ground-truthing of 245 independent, randomly selected test sites surveyed in the field [37].

### Measuring Species Co-occurrence at Camera Sites

We tested whether species co-occurred at camera sites by conducting hierarchical cluster analysis following McCune and Grace [40]. We defined each photographed species as either present (i.e., detected) or absent (i.e., not detected) at each camera site throughout the sampling period. We used a hierarchical agglomerative clustering strategy and Ward's linkage method with Euclidean distances to determine relatedness (i.e., a statistical index of co-occurrence) among species presence. To obtain a graphical representation of species co-occurrence, a dendrogram was produced with branches scaled with the percentage of information remaining in the analysis (i.e., the longer the branch lengths, the less the species at the dendrogram tips co-occurred).

We also tested for species co-occurrence at camera sites using nonmetric multidimensional scaling (NMS) of the actual species count data (i.e., a measure of relative abundance at a camera site, as opposed to using presence/absence only, as was done with hierarchical cluster analysis), a statistical approach that reduces data into fewer dimensions [41]. NMS is the best choice for reducing data that does not meet the assumptions of multivariate normality, and it is robust to large numbers of zero values [40], [42]. Thus, it is particularly appropriate for species counts, which are not normally distributed and contain zeros for rare species. Also following McCune and Grace [40], NMS ordination was conducted using the following parameters: Sorenson distance measure, 500 iterations, optimum number of dimensions identified by a change in stress value <5, and a Monte Carlo test run 250 times with randomized data. The software PCORD version 5.17 [43] was used for both hierarchical cluster analysis and NMS (see [44], [45] for algorithms used).

### Influence of Humans, Predators, Food and Habitat on Species Occurrence

Our study makes the reasonable assumption that predators might react to humans differently than prey [10], [12], [46]. Therefore, we could aggregate species counts at camera sites into a predators' guild (i.e., wolves, cougars, grizzly bears and black bears) and prey guild (i.e., moose, elk, white-tailed deer and mule deer). Coyotes were excluded from this analysis because they are considered meso-carnivores; they rely on smaller prey and accordingly did not associate strongly with the predator or prey guilds in this study (see Results).

We used regression tree analysis [47], [48], as a nonparametric approach to test whether humans, predators, food, and/or habitat influenced predator and prey species counts at camera sites. Covariates considered in the predator regression tree included humans (human counts at camera site), wild prey (sum of all prey species), cattle, and habitat at the camera site (elevation, forage and forest cover, measured in GIS, see above). Covariates considered in the prey regression tree included: humans, predators (sum of all predator species), cattle and habitat at the camera site.

Regression trees recursively partition the dependent variable (i.e., predator or prey count) into two comparatively homogeneous data clusters called nodes, and identify the independent covariate (i.e., humans, predators, food or habitat) that best explains the variation within each node. The optimum partition is determined by maximizing the LogWorth statistic (i.e., the negative base 10 logarithm of the p-value calculated from the sum of squares of the differences in means between the two groups formed by a partition [49]). Covariates in the regression tree can be re-used at each branch, thus non-linear relationships may be identified. We used the regression tree as an exploratory analysis; therefore we conducted recursive splitting of the tree to maximize significance until a minimum of five terminal groups was reached [40].

K-fold cross validation was used to assess regression tree model fit [47]. The dataset was divided into 10 randomly assigned bins of data. Regression trees were constructed using 9/10^th^ of the dataset and the remaining bin was kept aside. Predictions on species counts made by the regression tree were compared with data observed in the remaining bin and the process was reiterated 10 times. Fit was represented using R^2^ statistics. Regression tree analyses were conducted using JMP 7.0 software (SAS Institute^©^, Inc. 2007).

## Results

### Humans Co-occurred with Prey Species More than Predator Species

**Figure 1:**
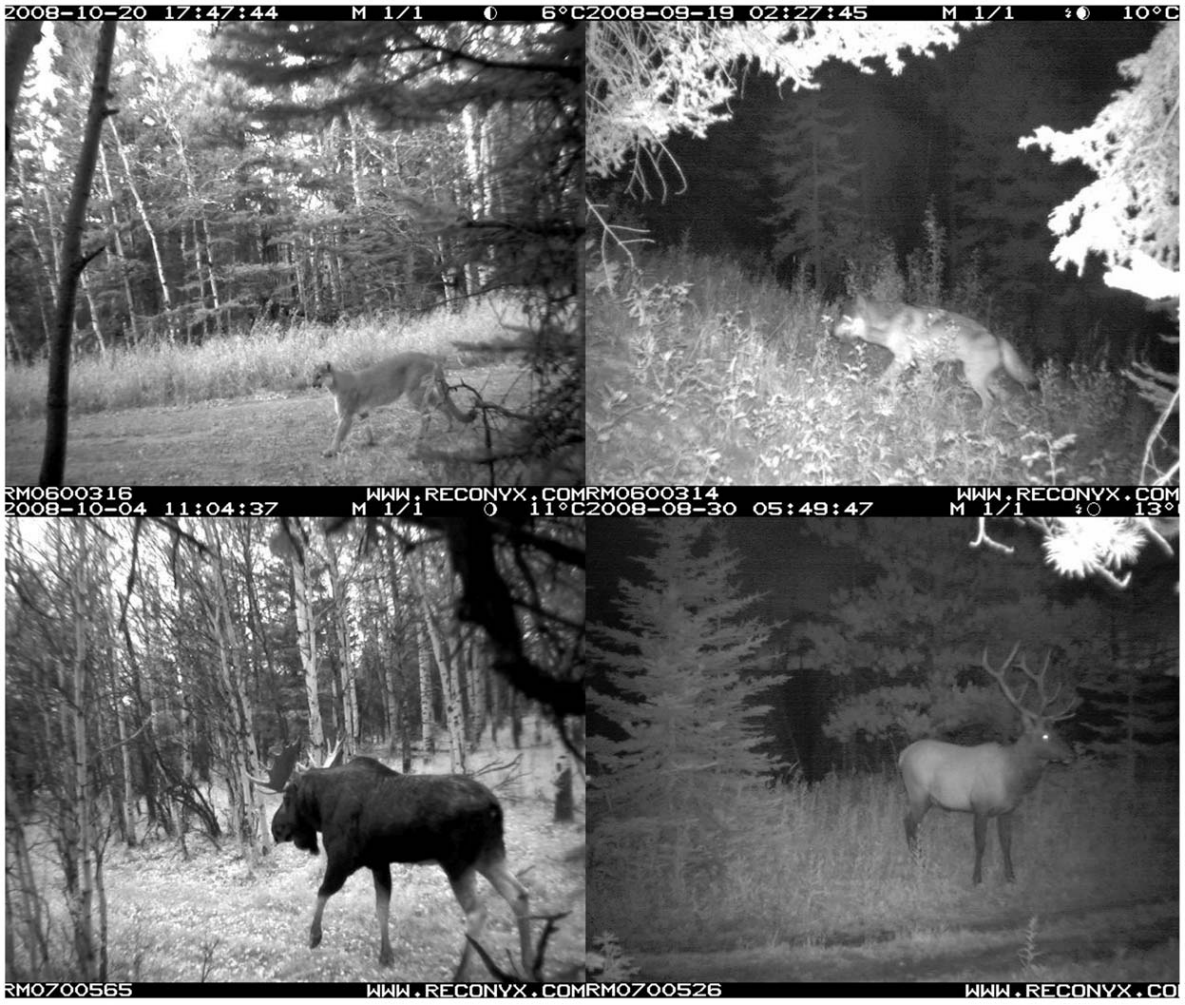
A sample of photos taken by cameras deployed on roads and trails in southwest Alberta, Canada during the summer of 2008. We photographed all large mammalian species in southwest Alberta, also including: cougar (top left), wolf (top right), moose (bottom left) and elk (bottom-right).

**Figure 2:**
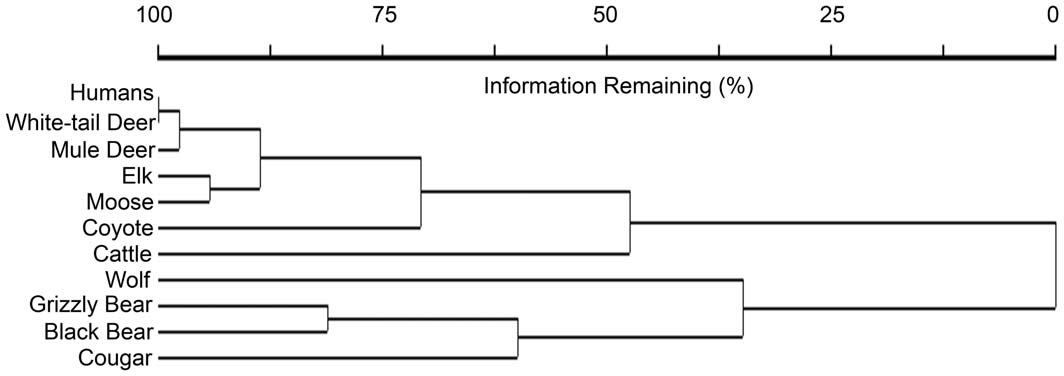
Dendrogram of the hierarchical cluster analysis of species presence/absence data that illustrates co-occurrence of species at camera sites in southwest Alberta, Canada during the summer of 2008. The dendrogram is scaled with the percentage of information remaining in the analysis, where less information remaining indicates a weaker association between species.

We obtained photographs (Fig. 1) of nine large mammalian wildlife species including wolves, grizzly bears, cougars, black bears, coyotes, moose, elk, mule deer and white-tailed deer, as well as humans and cattle. In Fig. 2, a dendrogram of the hierarchical cluster analysis of species presence/absence data illustrates co-occurrence of species at camera sites. Percent chaining of the cluster analysis was 23.08%. Predator species (i.e., wolves, grizzly bears, black bears and cougars) were distinct from the wild prey/human group (0% of information and the longest branches), indicating they did not typically co-occur at camera sites. Domestic cattle (47% of information remaining) and coyotes (71% of information remaining) were more closely clustered with wild prey/humans than predators. Humans were clustered with all wild prey species (indicated by short branches), including from lowest to highest association: elk and moose (89% of information remaining), mule deer (98% of information remaining) and white-tailed deer (100% information remaining).

**Figure 3:**
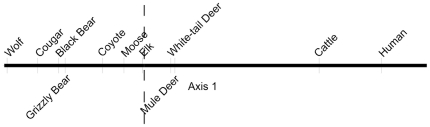
Co-occurrence of species at camera sites as determined by non-metric multidimensional scaling (NMS) ordination of species counts at camera sites in southwest Alberta, Canada during the summer of 2008. Ordinations along axis one are indicated. Location along axis one where the NMS score equals zero is indicated by a vertical dashed line.

Species ordination scores, illustrated along axis one of Fig. 3, indicate the relative co-occurrence of species at camera sites based on species counts at camera sites. The proportion of variance of the data represented by axis one was 0.737. We obtained a stress value of 5.703 and instability of <0.00001. On axis one, two species appear at the extremes: wolves (positive) and humans (negative). Other species of large predators (grizzly bears, black bears, and cougars) were placed close to wolves, indicating potential co-occurrence of the predator guild. At the other extreme, humans were most closely associated with domestic cattle. All prey species and coyotes were in the middle, yet closer to predators than to humans and cattle.

### Humans Co-Occurred with Prey but not Predator Species

**Figure 4:**
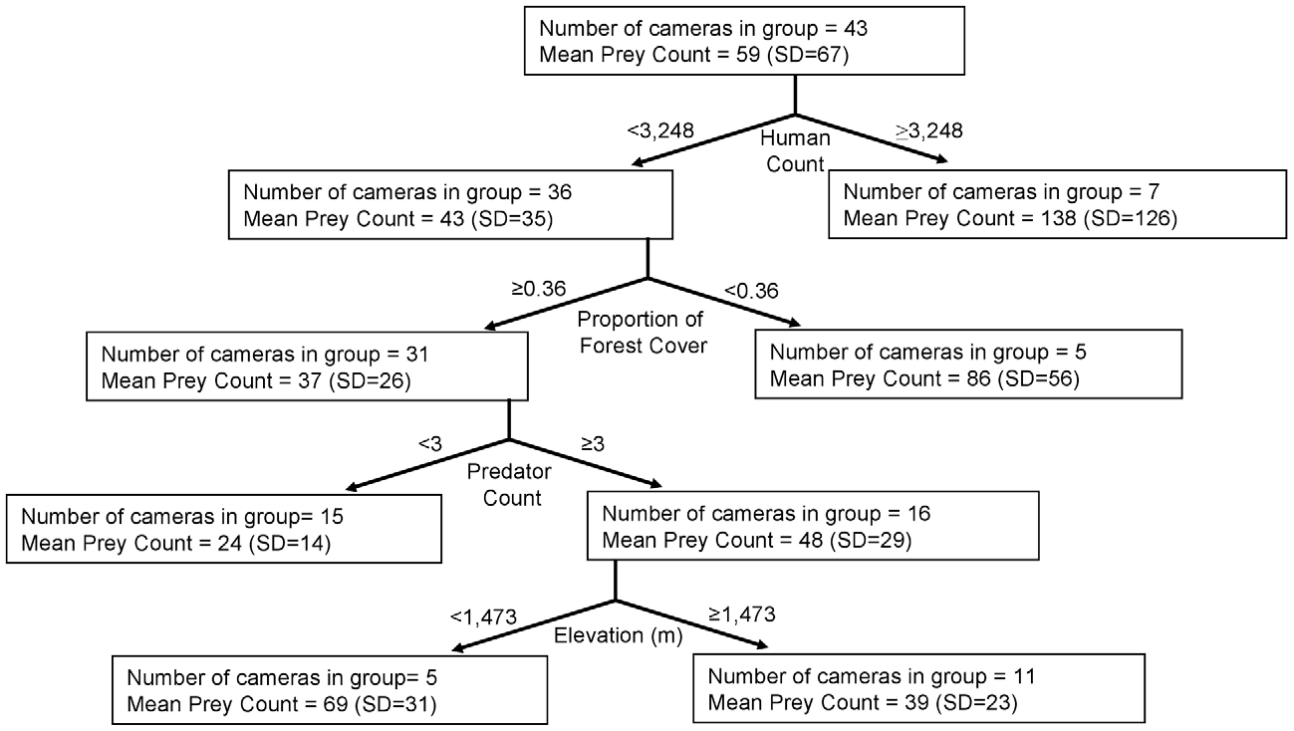
Regression tree analysis of large mammalian prey counts at camera sites in southwest Alberta, Canada during the summer of 2008. For each partition of the tree (indicated by arrows), the explanatory variable is shown with the value that best determines the partition (i.e., the cut-off point that maximizes homogeneity within a group). Indicated at each node are the number of cameras in the group and the mean number of prey photographs per 100 days (with standard deviation in parentheses).

The regression tree model of prey (Fig. 4) had a k-fold cross validation of R^2^ = 0.370. In general, prey were three times more abundant on roads and trails with >32 humans/day than on roads and trails with fewer humans. At the second level of the tree, on roads and trails with less people, prey were twice as abundant in less forested areas (i.e., where the percentage of forested area within 1 km of the cameras site was 36%) than forested areas. On the third level of the tree, in forested areas, prey were more abundant on roads and trails with ≥0.03 predators/day than roads and trails with fewer predators. Finally, on the fourth level of the tree, in forested areas with more predators, prey were more abundant at lower elevations (<1,473 m), than higher elevations.

**Figure 5:**
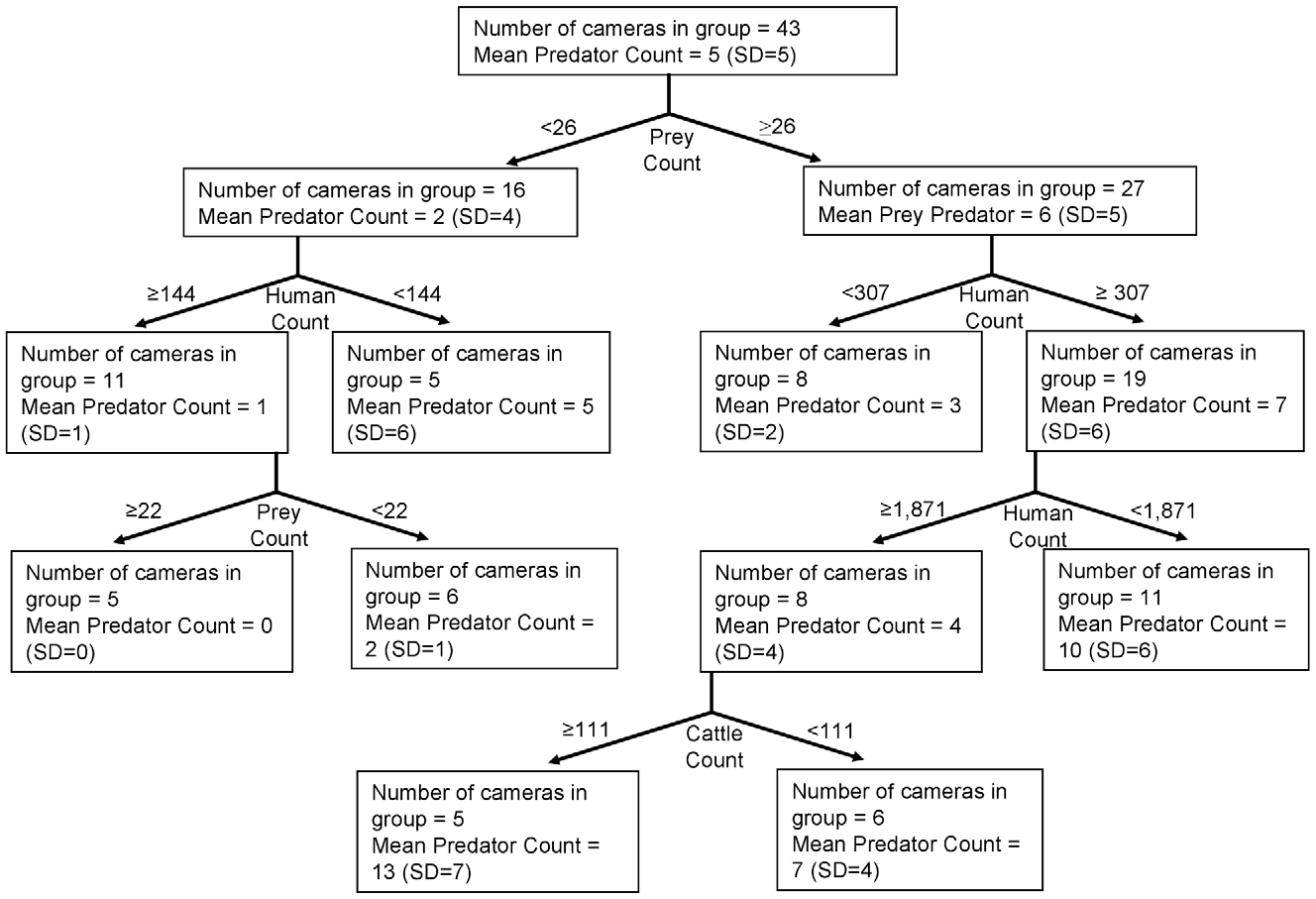
Regression tree analysis of large mammalian predator counts at camera sites in southwest Alberta, Canada during the summer of 2008. For each partition of the tree (indicated by arrows), the explanatory variable is indicated with the value that best determines the partition (i.e., the cut-off point that maximizes homogeneity within a group). Indicated at each node are the number of cameras in the group and the mean number of predator photographs per 100 days (with standard deviation in parentheses).

The regression tree model of predator count (Fig. 5) had a k-fold cross validation of R^2^ = 0.517. The first partition of the data indicated that predators were three times more abundant on roads and trails with ≥0.26 prey/day than on roads and trails with fewer prey. At the second level of the tree, on those roads and trails with more abundant prey, predators were more abundant if there were ≥0.31 humans/day on roads and trails compared with roads and trails with fewer humans. Also at the second level of the tree, on roads and trails with <0.26 prey/day, predators were more abundant on roads and trails with <1.44 humans/day than on roads and trails with more humans. However, at the third level of the tree, predators were less abundant on roads trails if there were ≥18.71 humans/day. Finally, at the fourth level of the tree, predators were more abundant on those roads and trails with <18.71 humans/day if there was ≥1.11 cattle/day, than on roads and trails with fewer cattle.

## Discussion

### Spatial Separation of Predator and Prey Species

We did not find predator and prey species aggregated together in the cluster dendrogram (Fig. 2) or ordination (Fig. 3). Rather, predator and prey species tended to co-occur with species of the same guild at camera sites. These results indicate partial spatial separation between predator and prey species and may suggest that prey are “winning” the predator-prey “space race” [1], i.e., prey species were more effective at avoiding predators than predators were at tracking prey. As a word of caution, predators may be selecting areas that improve their chance at capturing prey rather than areas with high prey density (e.g., [50]). In addition, predators may adopt unpredictable patterns in their use of space to increase prey uncertainty in perceiving predation risk of an area (e.g., [51]). These mechanisms, if present, limit the inference that prey are effectively avoiding being predated by just avoiding predators. However, the predator guild in this study represented diverse hunting strategies. Typically, cougars are solitary ambush predators, wolves are coursing predators that hunt in packs, and both black bears and grizzly bears are solitary, omnivorous species that hunt opportunistically [52]. It is therefore unlikely that all predators in this study selected similar habitats that likewise improved their chance at capturing prey, despite lower prey density there. Similarly, random patterns in the use of space by all predator species were not evident either. Therefore, we believe that spatial separation of predators from prey was likely the result of prey effectively avoiding predators.

### Humans Tip the Predator-Prey Space Race in Favour of Prey

Our results indicate that at high densities, humans might displace predators, providing a positive indirect effect on large mammalian herbivore species that are less sensitive to humans. Although prey were more abundant on roads and trails with more humans (i.e., >32 people/day), predators were less abundant on roads and trails that exceeded 18 humans/day, even if there were more prey there. Furthermore, telemetry data collected from wolves [19] and grizzly bears [53] in our study area confirm that predators avoid high-human use areas. Our results therefore support the hypothesis that humans can help prey win the predator-prey “space race”. Other studies also suggest that human disturbance that displaces predator species can benefit prey (e.g., [10], [12], [14], [46]). Similar mechanisms also appear to exist with regards to intraspecific competition. For example, human disturbance of dominant male grizzly bears can create refuges for female grizzly bears with cubs [54].

The positive association between herbivore prey and humans that we documented might not only be the result of humans displacing predators, but also due to humans improving forage around roads and trails [55]. High quality and quantity forage resources are correlated with high-human use roads and trails in the study area [19]. Humans might therefore provide the best habitat patches for herbivores by both deterring predators and improving food resources. However, high-quality forage habitat was not identified as a significant covariate in our regression tree analysis. Thus, we can reasonably conclude that herbivores likely used areas with high-human activity primarily as refugia from predators rather than for food resources.

Our results indicate species co-occurrence during the summer (i.e., April to November) only. The relative density and distribution of each species may potentially change during the winter. For example, predators may favour roads and trails during the winter for ease of travel if roads and trails are generally ploughed free of snow or snow is hard-packed by snow-machines (e.g., [56], [57]). Furthermore, humans hunt the herbivore species that we studied during parts of the winter, and thus areas of high-human density may not be effective refugia during that time. However, human hunters can displace herbivores onto private lands where hunting is not permitted by landowners [58]. Thus roads and trails with high-human density adjacent to private lands may be preferred by prey during the winter.

We acknowledge the possibility of finer scale shifts in space-use by predator and prey species in response to humans that could influence when and where predator-prey interactions occur on the landscape. For example, wolves are known to avoid areas of high-human density during the day, but use those same areas during the night when human activity is lower [8]. However, we could not detect any significant differences in predator and prey species occurrence at camera sites between day versus night in any of our analyses (Muhly 2010, unpublished data), which is why we chose whole-day analyses. Therefore, our data indicate that human activity had an effect on all predator and prey species throughout a 24-hour period.

Studies that measure resource distribution and predator and prey species use of space are rare (but see [59]), especially for large mammalian species, because of the difficulty in collecting data on their distribution. Our study indicates that camera traps and GIS technologies are useful to simultaneously document multiple species (including humans) use of space. Our study is unique, because in addition to resources and predators we considered the influence of humans on both predator and prey species use of space and thus we could document both trait-mediated direct and indirect effects of humans.

### Applications

We quantified a trait-mediated direct effect of human presence on predators (i.e., displacement) that had a trait-mediated indirect effect on prey species. The outcome of a predator-prey “space race” is often influenced by a spatial anchor, i.e., any environmental factor that is fixed in space that influences predator or prey fitness [1], [60]. Our results suggest that high-human use roads and trails might be a positive spatial anchor to prey, providing a spatial refuge from predators that are sensitive to human disturbance and potentially tipping the balance of the predator-prey space race in favour of prey. Although our study indicates such a mechanism, a greater understanding of the foraging strategies of each predator and prey species is worth investigating to determine if humans are also affecting the ultimate outcome of predator-prey interactions (i.e., predator and prey survival and fitness). In addition, we did not measure to what degree that human activity affects predator-prey interactions off roads and trails. However, large mammal prey species, for example elk, appear to be capable of detecting predators in areas in the tens of km^2^ in size (i.e., within drainages and home ranges; [38], [61]). We could reasonably expect that all large mammalian predators and prey in this study could detect humans within similarly sized areas. Thus, we believe the influence of humans extends off roads and trails. Finally, roads and trails with high-human activity (i.e., >18 humans/day; Fig. 5) occur throughout the study area [19]. Thus the effect of humans on predator-prey interactions has the potential to be pervasive.

Our results suggest that limiting human use of roads and trails to <18 humans/day could significantly reduce the effects on a large mammalian food web. However, wildlife managers should be aware that there are potentially several types and strengths of indirect effects of humans on food webs, of which providing prey refuge from predators is but one. For example, humans can also provide food for large mammalian prey by improving habitat [17], [18], which could have a positive indirect effect on predators by increasing availability of herbivore prey. Managers and scientists should consider and try to document a number of human influences on food webs when striving to effectively predict the consequences and mitigate the effects of human activities on ecosystem structure and function.

A growing human population and demand for ecosystem resources worldwide [62] suggests that effects of humans on food webs are likely to increase. Trait-mediated indirect effects of humans are increasingly documented in marine (e.g., [13], [63]) and terrestrial (e.g., [11], [58]) environments as well as at their interface (e.g., [14]). Much like how direct effects of predators on prey can have indirect effects on vegetation and biodiversity in general (e.g., [64]), the displacement of predator species by humans can potentially have indirect effects on interacting prey species that can ultimately have significant effects on the structure, function and biodiversity of an ecosystem. We therefore join others (e.g., [13], [14]) in encouraging scientists and managers to study, as we attempted in this research, both direct and indirect effects when assessing the influence of human activity on wildlife and ecosystems.
